# Thoughts on Regulating

**Published:** 1897-07

**Authors:** Howard E. Roberts

**Affiliations:** Philadelphia


### THOUGHTS ON REGULATING.[1]

BY HOWARD E. ROBERTS, D.D.S., PHILADELPHIA.

Mr. President and Gentlemen,—My excuse for writing this paper, if an excuse is necessary, is that I was asked to do so.

My reason for taking this subject is that I believe I correct irregularities somewhat differently from the majority, and with results gratifying to myself and apparently satisfactory to my patient. When I say gratifying to myself, I mean that I accomplish that which I attempt with the least worry about keeping things in place and without the necessity of having a laboratory

---

[1] Read before the Academy of Stomatology, Philadelphia, March 23, 1897.

handy. Satisfactory to my patient because there is the least amount of suffering with the greatest amount of comfort possible while going through an uncomfortable operation.

Apart from the annoyance of having the teeth sore, a great deal of irritation and worry is caused when anything is placed within the arch that either interferes with the free use of the tongue in talking or eating or that the tongue can play with or move. The patient is continually under a nervous strain and " mole hills seem like mountains," therefore I keep my appliances as much as possible upon the labial surfaces of the teeth.

Another great source of irritation comes from ligatures or bands either pressing against the gum or slipping out of place and working up around the neck of the tooth, therefore I see that it is impossible for them to get out of place. Keep things out of the way of the tongue and ligatures and everything else from touching the gum, and you have gone a long way towards making regulating bearable to the patient.

It seems almost unnecessary to say that all appliances should be made as small as possible. The appliances for nine-tenths of the cases of irregularity which are presented for correction can be made at the chair, and the only special instrument necessary is a jeweler's jam-plate for cutting threads. The jam-plate is not made mechanically to cut a thread, but, as its name implies, to jam or indent a thread upon a wire. It will do its work nicely and easily if care is taken to make the wire square where the thread is to be cut; to jam a thread upon a round wire is sometimes difficult.

A set of dies and taps can be procured, if it be desired, which will cut a thread perfectly; the wire or pin must be round, or nearly so, where these are used, and not square as in using the jam-plate.

In this set of four dies which I use the threads run 80, 100, 120, and 140 to the inch, and the one most used is 100. You can see the size of the wire upon which the thread is cut. With the dies there come taps to match. In using the jam-plate I make the tap I need from an old excavator.

I can best describe my mode of treatment by taking a case in practice, and have selected one where the teeth are somewhat jumbled. I do not attempt to do the work rapidly, but as comfortably as possible. In this case the corrected upper jaw is not a perfect arch, as the patient's parents did not care to carry it further, and the left lateral is a malformed tooth.

When this case was first presented an impression was taken in modelling compound of both jaws so as to show not only the teeth, but the gums over the roots of the teeth. I have never found it necessary to take the impression in plaster, and it would seem to be a waste of time. Always date and preserve the casts of the teeth as they are, before starting to make the correction, and study them carefully before going further or giving any advice.

An engagement was made on April 20 to make and put in place the appliance on the upper jaw. Previous to the patient coming, I provided a piece of platinum plate of thirty-two gauge to make bands from and a piece of half-round platinized gold wire which would reach from the first bicuspid on one side to the corresponding tooth on the other when resting upon the labial surface of the teeth, this made a spring bow. The ends were filed square for about three-eighths of an inch and threads were cut upon them with the jam-plate. To make the little nuts for the screw ends of the bow, the temper was drawn from an old excavator, the end filed square and the same thread was cut upon it, when it was re-tempered to a straw color; this made the tap. Holes were then drilled through a piece of heavy gold plate and tapped; small pieces were cut from the plate having the tapped holes in their centre, these were again run upon the tap to hold them and filed square, making them as small as possible for strength. This bow was made before the patient came. The canines required spreading, and with the patient in the chair a strip of platinum was cut one-eighth inch wide and three-eighths inch longer than the circumference of the tooth, bent in the form of a U and forced between the adjoining teeth and the canine with the ends out. With the finger hold the piece firmly against the palatine surface, then with flat-nosed pliers, which are smooth in the beak, grasp the two projecting ends, and, bearing upon the face of the tooth, close the pliers firmly, when you will produce a band closely fitting the tooth with the ends projecting at right angles, the bend being sharp and square. Cut one end off one-eighth inch from the band and the other one-fourth inch, bend the longer sharp, to fold over the shorter end and squeeze with the pliers, which clamps the ends together. Put a piece of pure gold over the joint inside the band and flow until the lap is thoroughly soldered. The annealing lamp will give heat enough. A band is thus made having a stout lug upon the face, through which drill a hole large enough to pass the end of the bow freely and file or grind away the excess of material to make a finished band and loop. This band was cemented to one canine and a similar band was made and fastened to the other.

The nuts being in place upon the bow, it is sprung into the bands and should rest upon the face of the most prominent incisor, which was ligated firmly to the bow and the nuts screwed against the loops on the bands; this was allowed to rest quietly for three or four days to allow the patient to become accustomed to it, after that the other incisors were either ligated to the bow or were drawn to it with rubber bands and the nuts were kept tight against the bands on the canines. On April 28 the same kind of an appliance was placed upon the lower jaw and both jaws straightened at the same time. I saw the patient nearly every day from that time until May 23, when she left the city; afterwards I saw her on June 10, 16, 26, and 27, July 10, 11, and 13, on which date I placed retaining bands on both upper and lower teeth, less than three months from the start, and the total time devoted to the case was twenty-nine hours. At no time did she complain of the pain, her gums were never inflamed, and she always came in smiling and seemed rather to enjoy the operation, as she was interested in it.

I have straightened teeth in the mouths of patients from seven years to forty years of age, and for ease and comfort I would take from fourteen to seventeen years, older rather than younger, and if a case is presented with the canines not erupted, as a rule, I would say wait until I can get bands upon them. If both parents and patient are not anxious to have the teeth put in place and be willing to assist and give the time to it, I would also say wait, they will probably want it done later when it will be easier for all, and particularly for the dentist. If asked how long it will take, I double the time I suppose will be necessary, and tell them that long; it is better to be through sooner than to take longer than they expect.

I believe a mistake is frequently made in thinking a case too easy and proper appliances are not made, the result being disappointment and failure.

In putting a bow in place it should rest naturally over the teeth near the gum and not spring towards the cutting edge; if it does, the tooth to which it is ligated may be elongated. If the ligature has a tendency to work off the tooth, drawing the bow down, make a band for the central and file a notch in the projecting lug, into which the bow will drop, and cement it to the tooth. To keep a rubber band or ligature from getting out of place, cement a platinum band near the tip of the tooth and tie the ligature just above the band; the band keeps it from slipping off and the tooth is larger above so that they cannot work up.

To make a band with a hook, over which to slip a rubber band or ligature, bend the lug over and file into shape. No metal band should be placed around a tooth without being thoroughly cemented. When a nut works loose, a ligature around the bow, over the nut, and hooped over the end of the bow and tied, will keep it in place.

I use the force of the screw, the spring of the bow, the elastic band, and the stress of a ligature tied tight all at once, or one at a time, as seems most desirable. It seems difficult to give good working advice, about a case from looking at the casts, because each case has an individuality of its own. The patient should be seen and known, and the ability of the dentist to carry out suggestions should also be taken into account.

For remuneration, I prefer to keep a record of all time spent upon a case and charge by the hour.

I do not like piano-wire for springs, or silver or German-silver appliances; they are unsightly; gold and platinum for a case does not cost much and are very much nicer.

I have found the vulcanite plate in the roof of the mouth an excellent thing upon which to become proficient in the use of profane language.

